# A Novel Method for Simulating the Extracellular Matrix in Models of Tumour Growth

**DOI:** 10.1155/2012/109019

**Published:** 2012-08-07

**Authors:** Alina Toma, Andreas Mang, Tina A. Schuetz, Stefan Becker, Thorsten M. Buzug

**Affiliations:** ^1^Institute of Medical Engineering, University of Lübeck, 23562 Lübeck, Germany; ^2^Centre of Excellence for Technology and Engineering in Medicine (TANDEM), University of Lübeck, 23562 Lübeck, Germany; ^3^Graduate School for Computing in Medicine and Life Sciences, University of Lübeck, 23562 Lübeck, Germany

## Abstract

A novel hybrid continuum-discrete model to simulate tumour growth on a cellular scale is proposed. The lattice-based spatiotemporal model consists of reaction-diffusion equations that describe interactions between cancer cells and their microenvironment. The fundamental ingredients that are typically considered are the nutrient concentration, the extracellular matrix (ECM), and matrix degrading enzymes (MDEs). The in vivo processes are very complex and occur on different levels. This in turn leads to huge computational costs. The main contribution of the present work is therefore to describe the processes on the basis of simplified mathematical approaches, which, at the same time, depict realistic results to understand the biological processes. In this work, we discuss if we have to simulate the MDE or if the degraded matrix can be estimated directly with respect to the cancer cell distribution. Additionally, we compare the results for modelling tumour growth using the common and our simplified approach, thereby demonstrating the advantages of the proposed method. Therefore, we introduce variations of the positioning of the nutrient delivering blood vessels and use different initializations of the ECM. We conclude that the novel method, which does not explicitly model the matrix degrading enzymes, provides means for a straightforward and fast implementation for modelling tumour growth.

## 1. Introduction

Cancer is one of the most common diseases in adulthood and has gained more and more attention in research of different scientific disciplines in recent years. We focus on malignant brain tumours such as gliomas, but the models presented in this paper are generic and can also be applied on different tumour entities and other solid tumours through variation of the corresponding parameters.

Generally, the models of tumour growth are described either on a macroscopic [1, 2], microscopic [3–5], or molecular level [6, 7]. Multiscale approaches, that is a combination of two [8, 9] or all three levels [10], are rarely used. The macroscopic models are generally based on continuous deterministic reaction-diffusion formalisms [1] and lead to a global description of the tumour. Thus, they depict a clinically significant size as it can typically be observed in magnetic resonance imaging or computed tomography. At this level the associating processes of tumour growth such as pressure and the therewith induced deformation of the environment tissue can be well observed and modelled. Such methods, though allowing for visual comparisons with medical, noninvasive in vivo imaging data, neglect the complex processes on the microscopic and molecular level. Mathematical approaches for simulating tumour growth on the cellular level are typically formulated in terms of discrete methods [11] like cellular automata [12, 13] or agent-based models [14, 15]. Nowadays hybrid modelling [4, 16–18] has become very prominent due to an improved description of the complicated processes by combining the continuous and discrete methods.

For understanding the in vivo behaviour of cancer, the fundamental challenge for mathematical simulation is the simplification of the underlying complex processes while maintaining realistic findings. Typically, a system of partial differential equations is used to model interactions between cancerous cells, the extracellular matrix (ECM), and matrix degrading enzymes (MDEs) that describe the haptotactic and/or chemotactic movement of the cells [16, 18–24]. For the ECM degradation and remodelling is considered. Degradation occurs where the proteolytic enzymes, such as the urokinase-type plasminogen activator (uPA) and matrix metalloproteinases (MMPs) are located. It has been proven that the degradation of the ECM occurs because MDEs are secreted by tumour cells (cf. [25]). To be able to model these complex interactions on a standard computer for a large number of cells it is inevitably necessary to develop a simplified description, that is, to identify processes that are of less significance for the overall spatiotemporal dynamics of cancerous cells. To this end, we introduce a novel simplified model describing the same processes as in the literature [16, 19–24], without explicitly modelling the MDE. We assume in our implementation that the cancer cells themselves are able to degrade the matrix. This assumption is motivated by the fact that the tumour cells are overexpressing the responsible enzymes. To get similar results, we determine the level of the degradation on the basis of a degrading parameter *α*_*f*_ and thus also the remodelling parameter *β*_*f*_. To validate the findings, we introduce different arrangements of the nutrient delivering blood vessels. Further, we compare the novel proposed model with common ones by simulating different initial conditions for the ECM. In this work, we do not consider cell-cell adhesion of cancerous cells not only due to the decreased expression of neural cell adhesion molecules (NCAM) in aggressive gliomas, such as the glioblastoma (GBM) [26] but also due to the focus on the haptotactic migration of cells through the extracellular matrix.

In this paper, we aim at simplifying the mathematical modelling of the complex biological processes and speeding computational time. In Section 2, we study the common method for cellular interactions and the simplest possible case. Finally, in Section 3, we discuss our results for different positions of capillaries and for a randomly or constantly distributed extracellular matrix, highlighting the successes of our approach. In Section 4 we conclude with a short outlook.

## 2. Materials and Methods

 For simulating brain tumour growth, we consider a two-dimensional area of brain tissue *Ω* = [0,1]×[0,1] which corresponds to 4 mm × 4 mm with boundary Γ : = ∂*Ω*. A 400 × 400 grid over *Ω* with a space step of *h* = 0.01 mm forms the basis for the discrete method since each square of the grid corresponds approximately to the area of a tumour cell, that is 6.25 × 10^−6^ cm^2^ (cf. [16]). The grid is introduced for computed tumour or necrotic cells but not for the host tissue, because of the clumps-like growth of the tumour. For interactions of the tumour with the host tissue, we recommend to have a look at the macroscopic models [27, 28]. At this level one achieves a better representation of processes like deformation of the environmental host tissue.

### 2.1. Hybrid Model

The hybrid model (cf. [4]) is composed of a continuous and a discrete part. For modelling tumour growth, a discrete description is used to account for the motion of individual cells. The remaining factors are based on partial-differential equations. For the extracellular matrix it has been assumed to be directly affected by the cancerous tissue. To this end, the complete system of equations consisting of the distribution of cancer cells *c*, nutrient concentration *u* and extracellular matrix (ECM) density *f* is given by:

(1a) ∂c∂t=Dc∇2c−χ∇·(c∇u)−ρ∇·(c∇f),

(1b) ∂u∂t=Du∇2u−αuuc,

(1c) ∂f∂t=−αffc+βff,

in *Ω* × [0, *T*], where *T* defines the end of a given time interval, *D*_*c*_ and *D*_*u*_ denotes the diffusion coefficients of the tumour cell and nutrients, respectively. Furthermore, *χ* is the chemotaxis coefficient and *ρ* the haptotaxis coefficient. Uptake and decay of particular substances due to tumour growth are *α*_*u*_ and *α*_*f*_ (uptake rate for nutrients and ECM), *β*_*f*_ represents the remodelling parameter for the ECM.

To compare our results with common ones, we outline also the approach for modelling the extracellular matrix effected by enzymes. The complete system including the matrix degrading enzymes' (MDEs) concentration *m* is defined in *Ω* × [0, *T*] as follows:

(2a) ∂c∂t=Dc∇2c−χ∇·(c∇u)−ρ∇·(c∇f),

(2b) ∂u∂t=Du∇2u−αuuc,

(2c) ∂f∂t=−α~ffm+β~ff,

(2d) ∂m∂t=Dm∇2m+βmc−αmm,

where *D*_*m*_ denote the diffusion coefficients of the enzymes, *α*_*m*_ the decay coefficient, and *β*_*m*_ represents the production constant for MDEs.

Due to the diffusive behaviour of the MDEs, the values are different for the uptake and the remodelling of the extracellular matrix comparing to (1c). Hence, we label them for the system ((2a), (2b), (2c), (2d)) ${\hat{\alpha }}_{f}$ and ${\hat{\beta }}_{f}$, respectively.

**Figure 1:**
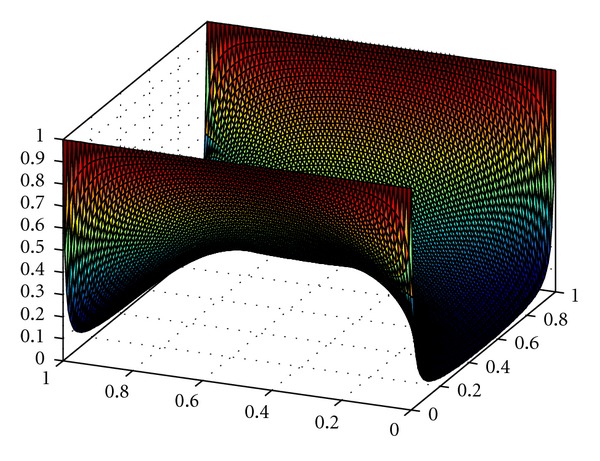
Initial distribution of the nutrients for blood vessels placed at two parallel boundaries.

For the initialisation of both models 441 tumour cells are placed in the middle of domain *Ω*. As for the different initial conditions for the ECM we refer to Section 2.3. The amount of initially available nutrients is estimated from the solution of the equation:

(3) $$\begin{array}{c} -D_{u}\nabla^{2}u+\alpha_{u}uc_{\text{ini}}=1, \end{array}$$

where *c*_ini_ represents the initial tumour with 441 cells in the middle of the domain. This equation denotes the availability of nutrients in the cerebrospinal fluid (CSF), which is a supplier of essential nutrients to the brain [29]. The initial nutrient concentration for two parallel blood vessels is shown in Figure 1. Depending on the location of the blood vessels, we have Dirichlet and Neumann boundary conditions (see Section 2.3).

The initial concentration for MDE is set to zero throughout the domain. For the concentration of the MDE and ECM we assume zero flux boundary conditions. 

### 2.2. Nondimensionalisation

 To obtain a similar magnitude in the range [0,1] for all computed quantities, we rescale and nondimensionalise the variables and parameters of the proposed method ((1a), (1b), (1c)) and the common approach ((2a), (2b), (2c), (2d)). The dimensionless variables are defined as:

(4) x^=xL,  t^=tτ,  c^=cc0,

(5) u^=uu0,  f^=ff0,  m^=mm0.

For a more detailed description we refer to Table 1 For the appropriate length scale *L* we use 0.1 cm (taken from [19, 23]), for the time *τ* = *L* ^2^/*D*, where *D* = 10^−6^ cm^2^/s is a representative diffusion coefficient [23]. For the tumour cell density *c*_0_, the nutrient concentration *u*_0_ and ECM density *f*_0_ following [16], the matrix degrading enzyme density *m*_0_ = 0.1 nM is taken from [19].

 For the dimensionless cell diffusion coefficient of the tumour cells we get ${\hat{D}}_{c}=\tau D_{c}/L^{2}=10^{-5}$ and for the dimensionless haptotaxis parameter $\hat{\rho }=\tau \rho f_{0}/L^{2}=0.26$ [16, 23]. We assume the chemotactic parameter *χ* to be equal to the haptotactic parameter, so that the cells are equally attracted to nutrients and to interact with the ECM. The parameter *D*_*u*_ = 10^−5^ cm^2^/s is taken from [16], so we get ${\hat{D}}_{u}=\tau D_{u}/L^{2}=10$. The uptake rate is assumed to be ${\hat{\alpha }}_{u}=\tau c_{0}\alpha_{0}/u_{0}=6.25\cdot 10^{-5}$. The parameters of the MDE, ${\hat{\beta }}_{m}=1$, and ${\hat{\alpha }}_{m}=0$ are taken from [16]. The dimensionless diffusion coefficient of the MDE is assumed to be 0.08. For the MDE dependent model ((2a), (2b), (2c), (2d)), we follow [30] for the ECM uptake ${\tilde{\alpha }}_{f}$ and the remodelling part ${\tilde{\beta }}_{f}$. Apart from that (system (1a), (1b), (1c)) we make a parameter estimation for *α*_*f*_ and *β*_*f*_ (see Section 2.4), since we are not aware of any values in the literature. For notational convenience, we drop the hats in the following.

### 2.3. Numerical Implementation

 For discretizing the systems of partial differential equations in ((1a), (1b), (1c)) and ((2a), (2b), (2c), (2d)) we use standard finite-difference and finite element method.

For computing the nutrients (1b), (2b), and the MDE (2d), we use the method of finite elements. Because of the discrete-continuum interaction in every time step, we have to solve the equations in the steady state. The boundary and initial conditions for nutrients depend on their position relative to the oxygen and glucose (nutrients) delivering blood vessels or capillaries. This can be modelled by placing them at all four surrounding boundaries, at two of them, or only on a single side. For the sites occupied by blood vessels we apply a Dirichlet boundary condition with a constant function *u*_*d*_. For the remaining boundaries we use zero flux boundary conditions (Neumann). Thus at any time *t* ∈ [0, *T*]:

(6) ∂u∂n=0 on⁡  ΓN,u=ud on⁡  ΓD,

where Γ_*N*_ and Γ_*D*_ are the Neumann boundary and the Dirichlet boundary, respectively, with Γ_*N*_ ∪ Γ_*D*_ = Γ. We set *u*_*d*_ = 1 since the concentration of nutrients, such as glucose and oxygen is highest in the capillaries.

For the tumour cell equations (1a) and (2a), respectively, we use the resulting coefficients of the five- and nine-point finite-difference stencil to generate the probabilities of the movement of an individual cell in response to its local milieu. The 5-point stencil is equivalent to the von Neumann neighbourhood and the 9-point stencil to the Moore neighbourhood. We implement both and use a switching mechanism to select one of them for each iteration and each cell (see Section 2.6). With *t* = *mk*, *x* = *ih*, and *y* = *jh* (*m*, *k*, *i*, *j*, *h* > 0), we use forward differences at time point *t*_*m*_ and second order central differences for the spatial derivative at point *x*_*i*,*j*_. The resulting equation for the 5-point stencil governing the chemotactic-haptotactic migration of a tumour cell in (1a) and (2a) has thus the form

(7) ci,jm+1=P0·ci,jm+P1·ci+1,jm+P2·ci−1,jm+P3·ci,j+1m+P4·ci,j−1m,

where *P*_0_ is proportional to the quiescent cells. *P*_1_, *P*_2_, *P*_3_, *P*_4_ are probabilities that are proportional to a movement of the cell to the right, left, up, or down, respectively. For the 9-point stencil, the resulting equation is straight forward:

(8) ci,jm+1=P0·ci,jm+P1·ci+1,jm+P2·ci−1,jm+P3·ci,j+1m+P4·ci,j−1m+P5·ci−1,j+1m+P6·ci+1,j−1m+P7·ci+1,j+1m+P8·ci−1,j−1m.

Here, we have additionally the probabilities *P*_5_, *P*_6_, *P*_7_, *P*_8_, which are proportional to a movement of the cell to the top left, bottom right, bottom left, and top right. For the extracellular matrix we model different initial conditions. On one side we take random values between 0 and 1, which corresponds to the density of the ECM [16]. On the other side, for a homogeneous behaviour a constant value *f*(*x*, *t*) = 0.8 is taken to allow for the assumption that the density of the ECM is high at the beginning, but smaller than 1, which would be equivalent to the maximal density and the value chosen in [16]. The choice of the value in this work is taken in reference to [31], where the extracellular space of gliomas has been described.

For solving the ECM equations (1c), (2c) we use Euler finite difference approximations. The density of the matrix is continuous, therefore a continuous description of the cells is needed. We use the tumour cell density *c* = 1 when a tumour cell is occupying the current location and take *c* = 0 otherwise. Intuitively, we use the highest value for the density, since the grid point is occupied by the cell. In this way we have a binary description of the tumour cells.

### 2.4. Parameter Estimation

 In this section we estimate the parameter *α*_*f*_ and *β*_*f*_ used in the newly proposed model, while the remaining parameters are taken from literature (c.f.  Section 2.2). In order to estimate the parameters and for the sensitivity analysis, we examine the behaviour of ECM *f* by varying *α*_*f*_ and *β*_*f*_ within a certain range.

The MDEs are diffusing, so that the concentration at positions where tumour cells are located is not so high as for the tumour cells, where we have the highest value *c* = 1. For the degradation parameter *α*_*f*_ of the extracellular matrix it is hence important to choose a value that is smaller than the value in (2c) which is 1. For the lower bound of the range, if the value 0 is included, in this case we will not have any degradations effects, which is not what we actually want. To this end, the parameter *α*_*f*_ is varying in the range (0,1).

The parameter ${\tilde{\beta }}_{f}$ is 0.015. Since the degradation parameter *α*_*f*_ is smaller than ${\tilde{\alpha }}_{f}$ and the equilibrium between degradation and remodelling of the ECM should remain valid, the upper bound of the remodelling parameter *β*_*f*_ will be 0.015. On that account and because 0 would mean no remodelling effects, the parameter is varying in the range (0,0.015].

When combining these two parameters, the stability has still to be ensured. Because of the explicit Euler method, which we use to solve the equation of the ECM and because the maximum of the cancer density *c* is 1, the stability requirement is

(9) $$\begin{array}{c} \left|1-k\alpha_{f}+k\beta_{f}\right|\leq 1, \end{array}$$

for *t* = *mk* and *c* → 1.

**Figure 2:**
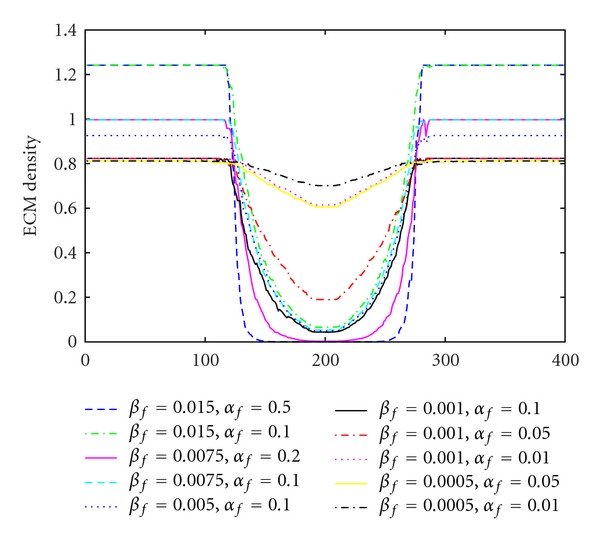
ECM profiles at *t* = 325 h for different *α*_*f*_ and *β*_*f*_.

We start by fixing the parameter *β*_*f*_ and vary *α*_*f*_, since the range of the remodelling parameter is much smaller. First *β*_*f*_ has to be chosen as high as possible, that is, *β*_*f*_ = 0.015. Now, we vary *α*_*f*_ just in a range of [0.02,1) for not breaking the stability requirement (9). The resulting ECM in the middle of the domain is shown in Figure 2. The density of the ECM is greater than 1, that is the production parameter is too big. For a value of *β*_*f*_ = 0.0075, the ECM density is approximately 1, and for *β*_*f*_ = 0.005 the ECM is 0.92 (see Figure 2). At last the production parameter was chosen to be *β*_*f*_ = 0.001 and *β*_*f*_ = 0.0005. For each value for *β*_*f*_ mentioned, we tested different values for *α*_*f*_, whereas *α*_*f*_ was selected w.r.t. *β*_*f*_ with the aim to archive a realistic, not too big production of the ECM and a degradation of the matrix, so that the cells can become invasive. A selection of different combinations of *α*_*f*_ and *β*_*f*_ as well as the resulting ECM *f* are given in Figure 2. The values for which the best equilibrium is archived are *β*_*f*_ = 0.001 and *α*_*f*_ = 0.01 (see Figure 2). These values reproduce the tumour growth extremely well (see Figure 8) throughout this work. 

### 2.5. Cell Actions

 The tumour equations (1a) and (2a) describe solely the motility of the cells. For modelling the cell actions proliferation, death and quiescence we consider the following criteria. In each time step and for each tumour cell we account for the local nutrient concentration and decide based on this how the cell will react. In case that the nutrient value for the respective cell is under a critical threshold *u*_crit_ = 0.4, we assume that the cell will die due to insufficient nutrients. Consequently, the cell is marked as necrotic tissue. In contrast to [16] the necrotic cells are not considered for the next step, since this material is not degraded by the macrophages of the brain (microglia) like it is the case for the apoptotic material due to phagocytosis [32]. Having checked the necrosis criterion, each cell moves according to the scheme described in Section 2.3. In case the nutrient concentration is high enough, that is, *u*_crit_ ≥ 0.4, the cell is selected to divide. The duration of the cell cycle is in general one day [6], in our case we assume that the proliferation takes eight hours, which is realistic for malignant tumours [16].

The grid location onto which the daughter cells are placed depends upon the cells occupying the neighbourhood of the mother cell. One daughter cell will always replace the mother cell, which is common to do so [16, 33]. Instead to place the second daughter cell randomly as usual [16], we placed it chemotacticially, that is in the nontumorous and nonnecrotic location with the highest nutrient concentration (if there is more than one free) in the neighbourhood of the mother cell. The choice of the neighbourhood is described in Section 2.6. This chemotacticially placement is also valid for the movement of a tumour cell.

In case there is no free space in the neighbourhood, the tumour cell becomes quiescent until free space is available or the cell becomes necrotic due to insufficient nutrient [16, 18, 34, 35]. Of course one cell can also get into a quiescent state because of the migration scheme.

Invasive glioma cells are resistant against apoptosis [36, 37] in favour of the cell survival. Furthermore, [38] proved by means of in vitro experiments that apoptosis is suppressed in GBM, hence we do not consider apoptosis in the proposed model in contrast to [16].

### 2.6. Neighbourhood and Update

 The choice of the local neighbourhood of an individual cell is crucial for the update of its state for lattice-based models [39–41]. To avoid unrealistic pattern formation, we use a novel effective and simply method [11], which introduces a fifty-fifty chance to decide if we take the eight neighbours into account or the four orthogonally surrounding cells. For each cell, at each discrete time point we generate a random number to decide whether the Moore or the von Neumann neighbourhood is favoured.

Another problem can arise from the strategy for updating the state of a tumour cell inside the lattice. If we would run sequentially to look at every tumour cell one by one, the first cell has often more possibilities for migration (in case of division: to place the daughter cells) than the one being located very next to it. Consequently, cells are not updated in a left-to-right or top-to-bottom manner but randomly [16].

## 3. Results and Discussion

**Figure 3:**
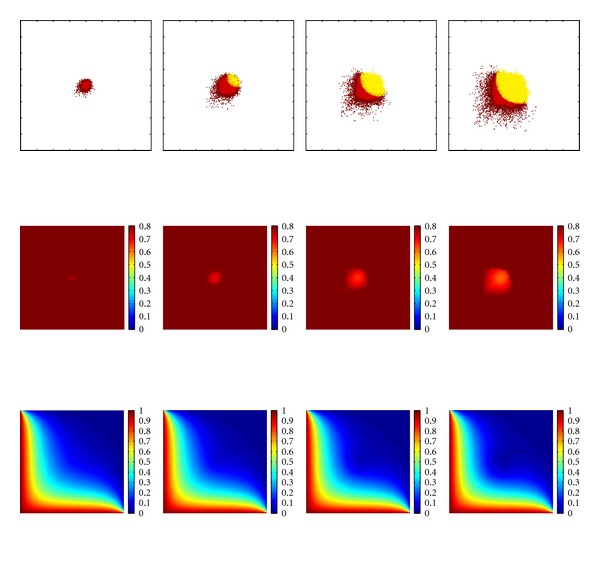
Simulation results for the proposed model ((1a), (1b), (1c)) at points in time *t* = 50 h, 150 h, 250 h, and 350 h. The initial condition of the nutrients is given by the blood vessel placed at the left side and at the bottom of the domain and are shown in the third row. The extracellular matrix is initialised with a constant value and is illustrated in the second row. For the cancerous cells in the first row light grey represents necrotic tissue, dark grey quiescent cells and black proliferating and migrating cells. The colouration for the remaining data is given in the colour bar.

**Figure 4:**
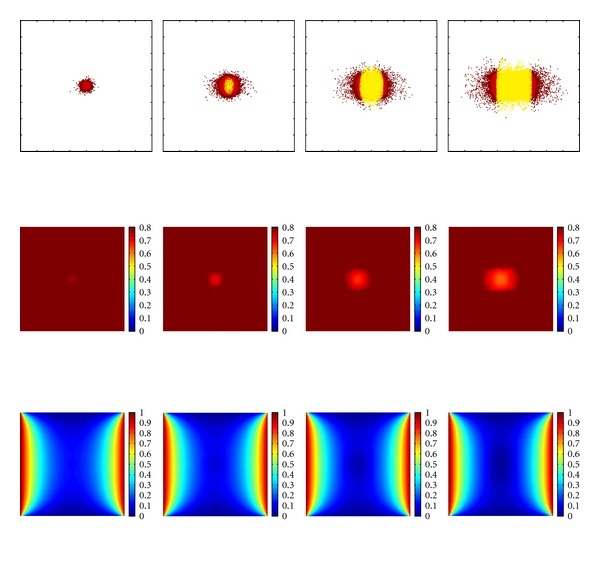
Simulation results for the proposed model at points in time *t* = 50 h, 150 h, 250 h, and 350 h. The initial condition of the nutrients is given by the blood vessel placed at the left and right sides of the domain and are shown in the third row. The extracellular matrix is initialised with a constant value and is illustrated in the second row. The first row is showing the distribution of the cancerous cells. Colouration as given in Figure 3.

The distribution of the extracellular matrix, the progression of nutrients, and the tumour cell arrangement over time are shown in Figures 3 and 4. There, the obtained tumour distributions are illustrated for up to 700 iteration steps, which is equivalent to a period of time of 350 h. At this point in time the tumour is hypoxic, that is, the mean value of nutrients and especially of the oxygen concentration is under a nondimensional value of *u* = 0.5. With hypoxia the tumour growth will transfer into the vascular stage through the process of angiogenesis [42].

In a first step, a homogeneous ECM (see also Section 2.3) and two neighbored blood vessels at the left side and at the bottom of the domain has been assumed. The results are illustrated in Figure 3. An increasing size of the tumour can be observed over the time. The steep rise can also be observed in Figure 8, bottom right. Likewise the distinct behaviour of the invasive cells migrating in the direction of the higher nutrient concentration is clearly visible and a large necrotic region. The nutrients are shown in the bottom row in Figure 3. The big consumption rate of the tumour cells is notable visible especially in the middle of the domain where the nutrient concentrations becomes smaller over time. The density of the ECM is shown in the middle row of Figure 3. The shape of the degradation is similar to the shape of the tumour cells. The boundary of the degraded matrix is less apparent, this is because individual tumour cells can not degrade the matrix just by moving through it just one time. In its centre, the ECM has been degraded more. There, the tumour bulk is located.

Computer simulations using two parallel blood vessels and a constantly initialized extracellular matrix is shown in Figure 4. The distribution of the tumour is invasive and a necrotic region due to the lack of nutrients (lower row) can be observed. Once more, the close interaction of tumour cells (upper row) and ECM (middle row) is clearly visible by means of the similar shape of both.

**Figure 5:**
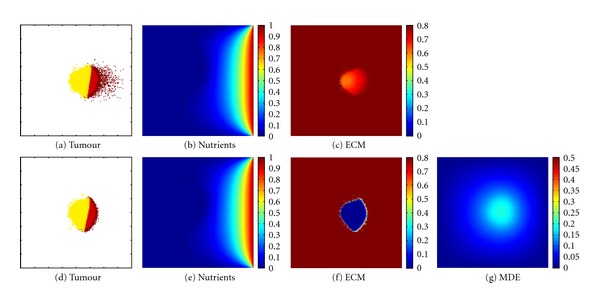
Simulation results (a–c) of the proposed method ((1a), (1b), (1c)) and (d–g) of the common method ((2a), (2b), (2c), and (2d)), respectively, at time *t* = 375 h. The initial condition of the nutrients is given by the blood vessel placed at the right side of the domain. The extracellular matrix is initialised with a constant value. Colouration as given in Figure 3.

The simulated tumour depicts a distribution that can typically be observed in vivo A large necrotic core surrounded by a rim of quiescent cells and an outer rim of strongly diffusive glioma cells. As a further step and to show the importance of the novel method, we compare results of the proposed approach with results of common ones. To this end, computer simulations using system ((1a), (1b), (1c)) and ((2a), (2b), (2c), (2d)) have been run. In a first step, one nutrient delivering blood vessel at the right side of the domain has been assumed. Given a homogeneous extracellular matrix distribution the results are shown in Figure 5. Comparing these data, differences for the ECM in Figures 5(c) and 5(f) can be observed, since the assumption for degradation of the matrix is different. The extracellular matrix modelled on the basis of system ((2a), (2b), (2c), (2d)) has been reduced through the diffusive MDEs shown in Figure 5(g) and the matrix simulated with the new introduced system ((1a), (1b), (1c)) is degraded by the tumour cells shown in Figure 5(a). 

For the tumour cell distribution a quite similar size of the bulk can be observed, however, there are differences concerning the invasive properties of the cells. For the tumour progression modelled with the proposed approach ((1a), (1b), (1c)) the invasive properties of the cells are more distinct. This is indeed characteristic for malignant tumours and consistent with in vivo and in vitro glioma experiments (cf. [37, 43] and references therein). However, this feature cannot be seen in Figure 5(d). The novel introduced method seems to capture this behaviour better. As expected, the nutrient progression is similar in all figures, since these are modelled in the same way in both models.

**Figure 6:**
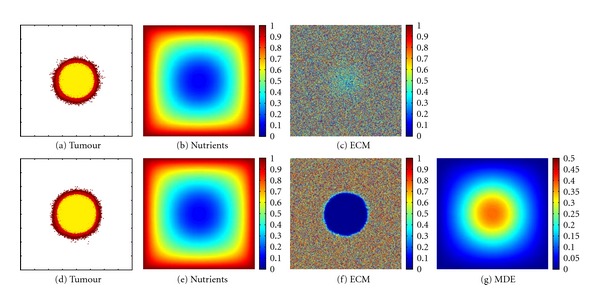
Simulation results (a–c) of the proposed method ((1a), (1b), (1c)) and (d–g) of the common method ((2a), (2b), (2c), (2d)), respectively, at time *t* = 375 h. The initial condition of the nutrients is given by blood vessel placed at all sides surrounding the domain. The extracellular matrix is initialised with random values (heterogeneous). Colouration as given in Figure 3.

**Figure 7:**
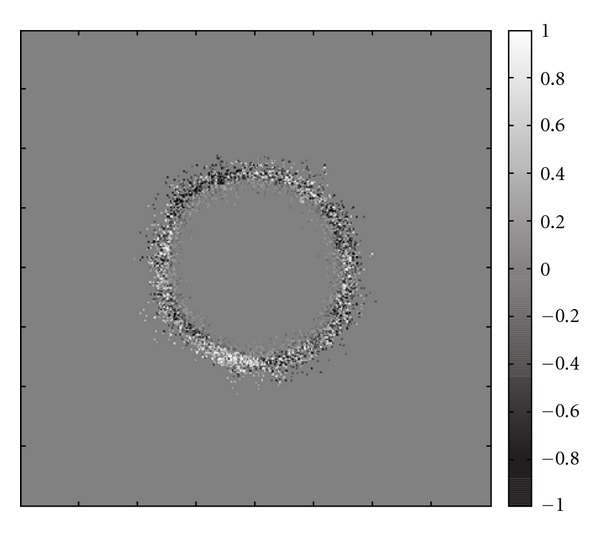
Difference image of the tumour cell distributions (Figures 6(a) and 6(d)) computed with the two described methods at time *t* = 375 h.

At last, we compare the tumour distribution assuming the typical Dirichlet boundary conditions for the nutrients, that is, we assume blood vessels located at all boundaries of the domain. Furthermore, a randomly initialised ECM (Figures 6(c), and 6(f)) is assumed. The tumour cell arrangement is shown together with the nutrients, ECM, and MDE in Figure 6. We notice a quite similar structure of the tumour cells (Figure 6(a)) simulated with the proposed method ((1a), (1b), (1c)) and the tumour cell distribution shown in Figure 6(d) modelled using ((2a), (2b), (2c), (2d)). There are only subtle differences, that have to be attributed to the probabilistically controlled model. For a better comparison of the differences of the tumour cell distributions obtained using ((1a), (1b), (1c)) and ((2a), (2b), (2c), (2d)), the difference image of Figures 6(a) and 6(d) is given in Figure 7. Apart of a small rim, only zero entries occur. The white points, representing a value 1, and black points (with values −1) show the missing tumour cells in either of the two images, the entries coloured with grey denote different states of the cancerous cell (proliferating, quiescent, necrotic). As mentioned before, this must not be misunderstood as a quantitative error difference or estimation because of the probabilities of cell movement and the randomly-guided model (see Section 2).

**Figure 8:**
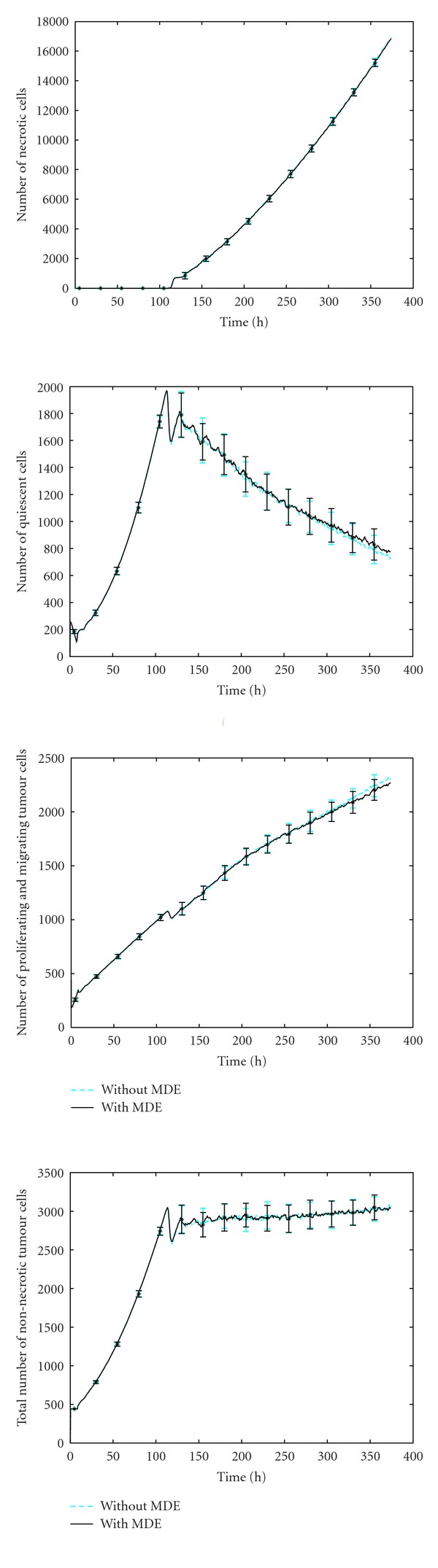
Average number of necrotic cells (upper row, left), quiescent cells (upper row, right), proliferating and migrating tumour cells (lower row, left), and total number of nonnecrotic tumour cells (lower row, right) over time with the respective standard error.

However, we have simulated the lastly described model (cf. Figure 6) one hundred times with different random number seeds and show the average behaviour of the different cells with the respective standard error for both methods (the proposed method ((1a), (1b), (1c)) and the common method ((2a), (2b), (2c), (2d))) in Figure 8. For the distribution of the necrotic cells shown in the upper left figure the differences are hardly visible. Only after approximately 110 h small differences can be observed for the progression of the quiescent tumour cells (upper right figure). The same behaviour can be seen in the lower two plots, where the distribution of the migrating/proliferating and all tumour cells, including quiescent cells are displayed. These highly similar results indicate the potential for the simplified method without explicitly simulating the matrix degrading enzymes. At this, another important advantage of the introduced model is the computation time (Table 2). The proposed model ((1a), (1b), (1c)) takes about 2.5 less time compared to model ((2a), (2b), (2c), (2d)) in case the ECM is initialized with a random distribution (Table 2). This reduction in run-time does even increase up to a factor of 6.3 in case the ECM is initialized to a constant value and just one blood vessel is assumed. Simulations have been carried out using a single-threaded MATLAB implementation and have been run on a Pentium i7920 with 2.67 GHz and 12 GB of RAM.

## 4. Conclusions and Outlook

 A hybrid approach has been developed that uses a coupled continuum-discrete model to simulate tumour growth. This method is devoted to the modelling of cellular processes of tumour cells, which is itself part of a complex system. The availability of efficient approaches therefore is an essential prerequisite for modelling tumour growth. To this end, a novel lattice-based approach has been developed that does not only provide a significant simplification compared to previous models, but also is computationally efficient (Table 2) and, moreover, depicts a more invasive behaviour of tumour cells, which is an important character of gliomas.

The degradation of the ECM in the area covered by the tumour can clearly be seen in all simulation results. This behaviour confirms our hypothesis of the direct interaction between tumour cells and the extracellular matrix. As an important feature the novel method provides the best results using a constant matrix, where the invasive behaviour of tumour cells can clearly be observed, in accordance to in vivo and in vitro behaviours [37, 43].

Furthermore, the proposed model requires about 2.5 less computation time compared to the common model in case the ECM is heterogeneous (Table 2). This reduction in run-time does even increase up to a factor of 6.3 in case the ECM is chosen to be homogeneous (assuming one blood vessel).

Comparing the results for modelling haptotactic-chemotactic cancer growth using the common choice of the environment and the novel introduced approach, a rather similar size of the tumour can be observed, in case a heterogeneous ECM is used. Faced with the requirement of modelling much more complex processes than the degradation of the ECM through the MDE (in order to adequately model tumour growth) the proposed model provides a reasonable tradeoff between complexity and accuracy.

Prospectively, it will also be essential to extend the devised model by cell-cell interactions such as pressure on neighbouring cells caused by mitosis or cell migration. We expect, that this pressure will make the thickness of the proliferating rim more distinct [44]. For a more realistic description of the in vivo processes, incorporating the immune system [45] and effects of therapies [46, 47] forms the central focus of our current work. In this case, the established efficiency gain might pay off even more through alternative numerical solvers than the implemented finite element method. Further, we aim at devising multiscale tumour growth models that not only account for cell-cell interaction but also for molecular events as well as for information available from the macroscopic embedding. The important effects of, for example, epidermal growth factor receptor (EGFR) which are overexpressed in gliomas [48] on the molecular level have to be included.

## Figures and Tables

**Table 1:** Variables used in the hybrid model: The variable of the dimensional system ((1a), (1b), (1c)) and ((2a), (2b), (2c), (2d)), a description, their units, the computation for the corresponding nondimensional parameters and references.

Variable *y*	Description	Unit	Formula of $\hat{y}$	Reference
*c*(**x**, *t*)	Cancer cell	Cells cm^−3^	*c*/*c*_0_	[16, 23]
*u*(**x**, *t*)	Nutrient concentration	M O_2_ cm^−3^	*u*/*u*_0_	[16, 23]
*f*(**x**, *t*)	ECM	M	*f*/*f*_0_	[16, 23]
*m*(**x**, *t*)	MDE	M	*m*/*m*_0_	[16, 19, 23]

**Table 2:** Computation time in minutes for the common model ((2a), (2b), (2c), (2d)) and the proposed approach (without explicitly modeling MDE, ((1a), (1b), (1c)) at different configurations.

Initial ECM	Capillaries	Standard model	New model
	All sides	85	32
Random	Two sides	125	48
	One side	138	61

	All sides	92	28
Constant	Two sides	133	31
	One sides	151	24
